# Supplementary material for: Awareness and Knowledge of HPV, HPV Vaccination, and Cervical Cancer among an Indigenous Caribbean Community
Source: Int J Environ Res Public Health. 2022 May 7;19(9):5694. doi: 10.3390/ijerph19095694 (PMC9105034; doi:10.3390/ijerph19095694)
Supplement: Supplementary file 1 [file ijerph-19-05694-s001.zip › ijerph-1673966-supplementary.pdf]

PEAK Study

Participant ID: ________________ Date: ______________

1. **What is your age?**____________________

2. **What is your sex?**
   - ☐ Male
   - ☐ Female

3. **What do you do for work?**

   ____

4. **What is your marital status?**
   - ☐ Married
   - ☐ Living with partner
   - ☐ Divorced
   - ☐ Single
   - ☐ Widowed

5. **What is your race or ethnicity?**
   - ☐ **Kalinago**
   - ☐ **Black**
   - ☐ **Caucasian (White)**
   - ☐ **Mixed**
   - ☐ **Other:**____________________________

6. **What country were you born in?**

   ____

7. **What country were your parents born in?**

   ____

8. **Have you ever been diagnosed with any type of cancer?**
   - ☐ Yes
   - ☐ No

   If yes, what type of cancer were you diagnosed with?

   ____

9. **Has anyone in your family ever been diagnosed with cancer?**
   - ☐ Yes
   - ☐ No

   If yes, what type of cancer was your family member diagnosed with?

   ____

10. **What was this family members' relation to you?**
    - ☐ Grandparent
    - ☐ Parent
    - ☐ Spouse
    - ☐ Sibling
    - ☐ Child
    - ☐ Grandchild
    - ☐ Aunt/Uncle
    - ☐ Cousin
    - ☐ Other: ___________________________

11. **Do you know anyone else who has been diagnosed with cancer?**
    - ☐ Yes
    - ☐ No

    If yes, how do you know them?

    ____

12. **How many years of schooling have you completed?**
    - ☐ Primary school
    - ☐ High school
    - ☐ Some college
    - ☐ College graduate or higher

13. **Please circle the range your total household income for one year falls.**
    - ☐ Less than $5,000
    - ☐ $5,000 to 9,999
    - ☐ $10,000-19,999
    - ☐ $20,000-29,999
    - ☐ More than $30,000

14. **Do you have health insurance?**
    - ☐ Yes
    - ☐ No

15. Do you have a primary health care provider (e.g. Nurse, Doctor, District Medical Officer)
- ☐ Yes
- ☐ No

16. **If YES, have you seen your primary health care provider in the last year?**
- ☐ Yes
- ☐ No

17. What language do you speak?

____

Beliefs about Cancer

18. How likely are you to get cancer in your lifetime?
- ☐ Very likely
- ☐ Likely
- ☐ Somewhat likely
- ☐ Not likely
- ☐ Not likely at all

19. How worried are you about getting cancer?
- ☐ Very worried
- ☐ Worried
- ☐ Somewhat worried
- ☐ Not worried
- ☐ Not worried at all

20. What do you think about this statement: "There's not much I can do to lower my chances of getting cancer"
- ☐ Strongly agree
- ☐ Agree
- ☐ Somewhat agree
- ☐ Disagree
- ☐ Strongly disagree

21. Have you ever looked for information about cancer from any source?
- ☐ Yes
- ☐ No

22. If you have ever looked for information on cancer, where did you look for cancer information?
- ☐ Online newspapers
- ☐ Print newspapers
- ☐ Special health or medical magazines
- ☐ Internet
- ☐ Radio
- ☐ Local Television show
- ☐ National television show
- ☐ Health Center
- ☐ Other: ______________________

23. Do you think the following behaviors increase a person's chance of getting cancer…?

	Yes	No	Don't know
**A. Eating fruits and vegetables**			
**B. Smoking**			
**C. Exposure to the sun**			
**D. Being obese**			
**E. Having fair skin**			
**F. Having many sexual partners**			
**G. Having a family history of cancer**			
**H. Being a particular race or ethnicity**			
**I. Pollution exposure**			
**J. Radon exposure**			
**K. Talking a lot about cancer**			
**L. Spending time with someone who has cancer**			
**M. Experiencing high amounts of stress**			
**N. Drinking alcoholic beverages**			
**O. Not exercising regularly**			
**P. Eating a high fat diet**			

Colorectal Cancer - (cancer of colon or rectum)

24. Have you ever heard of colorectal cancer? You may have also heard this called colon, rectum, or bowel cancer.
- ☐ Yes
- ☐ No
- ☐ Don't Know

25. What do you think the likelihood is of you getting colorectal cancer?
- ☐ Very likely
- ☐ Likely
- ☐ Somewhat likely
- ☐ Not likely
- ☐ Not likely at all

26. Do you know how to get tested for colorectal cancer?
- ☐ Yes
- ☐ No
- ☐ Don't Know

27. DURING THE PAST 10 YEARS, have you had any test done for colon cancer? ***(Tests for colon cancer include stool testing such as Fecal Occult Blood Test (FOBT/FIT), colonoscopy, and sigmoidoscopy)***

- ☐ Yes
- ☐ No
- ☐ Don't Know

If yes, what type of test did you have?
- ☐ Stool Test (Fecal Occult Blood Test FOBT/FIT)
- ☐ Colonoscopy
- ☐ Sigmoidoscopy
- ☐ Don't know
- ☐ Other: ________________________

If yes, when?

____

28. How would you rate the importance of getting a colorectal cancer screening (colonoscopy/sigmoidoscopy or stool test such as FOBT/FIT test)?
- ☐ Very important
- ☐ Important
- ☐ Somewhat important
- ☐ Not important
- ☐ Not important at all

29. <u>If you haven't had a test for colon cancer in the last 10 years</u>, what is the ONE most important reason why?
- ☐ Too young
- ☐ No reason/never thought about it
- ☐ Didn't know I needed this type of test
- ☐ Doctor didn't tell me I needed it
- ☐ Haven't had any problems
- ☐ Put it off
- ☐ Too expensive/no insurance/cost
- ☐ Too painful, unpleasant
- ☐ Embarrassing
- ☐ Don't have a doctor
- ☐ Other: ___________________________
- ☐ Don't know
- ☐ Not applicable, I have had a CRC in the last 10 years

Other Cancer Questions

30. Have you heard of Human Papillomavirus or HPV? ***HPV is not the same as HIV.***
- ☐ Yes
- ☐ No
- ☐ Don't know

31. HPV is able to cause cervical cancer:
- ☐ True
- ☐ False
- ☐ Don't know

32. Most people have HPV at some point in their lives:
- ☐ True
- ☐ False
- ☐ Don't know

33. Before today, have you heard of the HPV vaccine (also known as the cervical cancer vaccine or Gardasil)?
- ☐ Yes
- ☐ No
- ☐ Don't know

34. If yes, where did you hear about the HPV vaccine? (check all that apply)
- ☐ Friends
- ☐ Family
- ☐ Healthcare professional (doctor or nurse)
- ☐ Television
- ☐ Radio
- ☐ Newspaper
- ☐ Internet
- ☐ Planned Parenthood
- ☐ Other: ________________________

35. The HPV vaccine has 1 dose:
- ☐ True
- ☐ False
- ☐ Don't know

36. How long has it been since you last visited a dentist or dental clinic?
- ☐ **I have never gone to a dentist or dental clinic**
- ☐ **Within the past year**
- ☐ **Within the past 2 years**
- ☐ **Within the past 5 years**
- ☐ **More than 5 years ago**

37. In general, would you say your nutrition habits are:
- ☐ Excellent
- ☐ Very good
- ☐ Good
- ☐ Fair
- ☐ Poor

38. How comfortable are you making an appointment, or visiting with a doctor or other health care providers?
- ☐ Very comfortable
- ☐ Comfortable
- ☐ Somewhat comfortable
- ☐ Not comfortable
- ☐ Not comfortable at all

39. In general, would you say your health is:
- ☐ Excellent
- ☐ Very good
- ☐ Good
- ☐ Fair
- ☐ Poor

40. Do you currently smoke?
- ☐ Yes
- ☐ No

WOMEN CONTINUE TO NEXT PAGE →

THIS SECTION FOR MEN ONLY

41. Have you ever received screening for prostate cancer?
- ☐ **Yes**
- ☐ **No**

42. If you responded "No" above, please explain why you have not received a screening for prostate cancer?

THANK YOU FOR PARTICIPATING!

We appreciate your responses!

THIS SECTION FOR WOMEN ONLY

Breast Cancer

43. What do you think the likelihood is of you getting breast cancer?
- ☐ Very likely
- ☐ Likely
- ☐ Somewhat likely
- ☐ Not likely
- ☐ Not likely at all

44. Do you know how to get tested for breast cancer?
- ☐ Yes
- ☐ No
- ☐ Don't know

45. How would you rate the importance of getting a mammogram?
- ☐ Very important
- ☐ Important
- ☐ Somewhat important
- ☐ Not important
- ☐ Not important at all

46. Have you had a Mammogram DURING THE PAST 2 YEARS?
- ☐ Yes
- ☐ No
- ☐ Don't know

47. <u>If you haven't had a mammogram in the last 2 years,</u> what is the ONE most important reason why?
- ☐ Too young
- ☐ No reason/never thought about it
- ☐ Didn't know I needed this type of test
- ☐ Doctor didn't tell me I needed it
- ☐ Haven't had any problems
- ☐ Put it off/laziness
- ☐ Too expensive/no insurance/cost
- ☐ Too painful, unpleasant
- ☐ Embarrassing
- ☐ Don't have a doctor
- ☐ Other: ___________________________
- ☐ Don't know
- ☐ Not applicable, I have had a mammogram in the last 2 years

How much do you agree with the following statements?

48. If someone hits my breast, I will get breast cancer.
- ☐ Strongly agree
- ☐ Agree
- ☐ Somewhat agree
- ☐ Disagree
- ☐ Strongly disagree

49. Women who have large breasts are more likely to get breast cancer than women who have small breasts.
- ☐ Strongly agree
- ☐ Agree
- ☐ Somewhat agree
- ☐ Disagree
- ☐ Strongly disagree

50. Breastfeeding a baby can protect you from getting breast cancer
- ☐ Strongly agree
- ☐ Agree
- ☐ Somewhat agree
- ☐ Disagree
- ☐ Strongly disagree

51. Are all breast lumps cancer?
- ☐ Yes
- ☐ No
- ☐ Don't know

Cervical Cancer Questions

52. **Have you heard of cervical cancer?**
    - ☐ Yes
    - ☐ No
    - ☐ Don't know

53. **Do you know what a Pap smear is?**
    - ☐ Yes
    - ☐ No
    - ☐ Don't know

54. **How often should you get a Pap smear?**
    - ☐ Only if you think you have cancer
    - ☐ Every year
    - ☐ Every three years
    - ☐ Every five years

55. **What do you think the likelihood is of you getting cervical cancer?**
    - ☐ Very likely
    - ☐ Likely
    - ☐ Somewhat likely
    - ☐ Not likely
    - ☐ Not likely at all

56. **How would you rate the importance of getting a Pap smear?**
    - ☐ Very important
    - ☐ Important
    - ☐ Somewhat important
    - ☐ Not important
    - ☐ Not important at all

57. **<u>If you haven't had a Pap smear in the last 3 years,</u> what is the ONE most important reason why?**
    - ☐ Too young
    - ☐ No reason/never thought about it
    - ☐ Didn't know I needed this type of test
    - ☐ Doctor didn't tell me I needed it
    - ☐ Haven't had any problems
    - ☐ Put it off/laziness
    - ☐ Too expensive/no insurance/cost
    - ☐ Too painful, unpleasant
    - ☐ Embarrassing
    - ☐ Don't have a doctor
    - ☐ Other: ___________________________
    - ☐ Don't know
    - ☐ Not applicable, I have had a Pap smear in the last 3 years

58. Please mark True or False…

	True	False	Don't know
**You cannot get a pap smear while on your period**			
**You need a pap smear even if you have not had sex**			
**You do not need a pap smear if you are not sexually active**			

<u>THANK YOU FOR PARTICIPATING!</u>

We appreciate your response
